# Adverse event profile of lomustine and temozolomide: a descriptive analysis from WHO-VigiAccess

**DOI:** 10.3389/fphar.2025.1534802

**Published:** 2025-03-06

**Authors:** Hua Luo, Shaohua Fan, Lu Liang, Youfu He, Jiangjie Chen, Chenghao Xu, Jing Zhu, Liwei Zhang

**Affiliations:** ^1^ Department of Orthopedics, Taizhou Hospital of Zhejiang Province, Zhejiang University, Taizhou, China; ^2^ Department of Operating Room, Taizhou Hospital of Zhejiang Province, Zhejiang University, Taizhou, China; ^3^ Department of Cardiology, Guizhou Provincial People’s Hospital, Guiyang, Guizhou, China; ^4^ School of Integrative Medicine, Nanjing University of Chinese Medicine, Nanjing, China; ^5^ Institute of Bone Metabolism, Taizhou Hospital of Zhejiang Province, Zhejiang University, Taizhou, China

**Keywords:** glioma, adverse drug reactions, WHO-VigiAccess, lomustine, temozolomide

## Abstract

**Introduction:**

In gliomas, various oncogenic factors can lead to an imbalance between cell proliferation and apoptosis. Lomustine inhibits tumor cell growth by disrupting DNA replication and repair mechanisms. In contrast, temozolomide, an imidazole tetrazine compound, promotes cell apoptosis through DNA alkylation. The present study aimed to systematically analyze and compare the adverse drug reactions (ADRs) associated with lomustine and temozolomide, as reported in the World Health Organization (WHO) VigiAcess database.

**Methods:**

Utilizing a retrospective descriptive analysis design, this study focused on two commercially available anti-glioma drugs. ADR reports pertaining to these medications were collected from the WHO-VigiAccess database. The data collection process involved gathering detailed information on various parameters, including age groups, gender, and geographical distribution of patients involved in the ADR reports. Additionally, the study examined the disease systems and symptoms reported alongside the adverse reactions, as recorded in the annual ADR summaries generated by the WHO. By calculating the proportion of adverse events reported for each drug, this investigation provided a comparative analysis of both the similarities and differences in the adverse reactions observed across the two anti-glioma drugs.

**Results:**

At the time of the search, a total of 22,854 adverse events (AEs) associated with the two anti-glioma drugs were documented in the VigiAccess database. Lomustine exhibits a higher reporting rate concerning blood and lymphatic system disorders, gastrointestinal disorders, and hepatobiliary disorders. In contrast, Temozolomide has a higher reporting rate for general disorders and administration site conditions, nervous system disorders, and skin and subcutaneous tissue disorders. The top five types of AEs for anti-glioma drugs are as follows: general disorders and administration site conditions (8,825 cases, 38.61%), blood and lymphatic system disorders (7,369 cases, 32.24%), gastrointestinal disorders (5,614 cases, 24.56%), nervous system disorders (5,047 cases, 22.08%), and investigations (4,855 cases, 21.24%).

**Conclusion:**

The present comparative observational study indicates that these inhibitors are associated with both common and specific adverse reactions, as documented in ADR reports. Clinicians should formulate individualized treatment plans that consider the adverse reactions linked to various drugs and the specific conditions of each patient, thereby promoting the rational use of these costly medications.

## Introduction

Glioma is the most prevalent primary intracranial tumor, accounting for approximately 30% of all brain and central nervous system tumors and 80% of all malignant brain tumors (Gusyatiner and Hegi, 2018; Xu et al., 2020). According to the current classification by the World Health Organization, gliomas are categorized into four histological grades, which can be further divided into low-grade (Grades I and II) and high-grade (Grades III and IV) tumors. Glioblastoma (GBM) is the most common high-grade glioma, constituting 45.2% of primary malignant brain tumors and central nervous system (CNS) tumors (Louis et al., 2021). GBM is an intractable disease, with a median survival of merely 15 months; only 5.5% of patients survive 5 years after diagnosis. In individuals aged 65 and older, the incidence rate has been shown to rise to 130 cases per million (Kanderi et al., 2024). The pathogenesis of glioma remains elusive; however, two identified risk factors include exposure to high doses of ionizing radiation and high-penetrance genetic mutations associated with rare syndromes. Additionally, carcinogenic factors such as nitrite-containing foods and viral or bacterial infections may also contribute to the development of glioma. The principal clinical manifestations of glioma include increased intracranial pressure, neurological and cognitive dysfunction, and epileptic seizures.

The highly aggressive nature of gliomas and their resistance to traditional treatments present significant challenges for patients. In gliomas, multiple oncogenic factors can create an imbalance between cell proliferation and apoptosis. Researchers are increasingly focusing on individualized treatment plans as their understanding of the biology of gliomas deepens. Among various treatment modalities, the chemotherapy drugs lomustine and temozolomide have garnered considerable attention due to their notable efficacy in glioma treatment. Lomustine is a nitrosourea and an oral alkylating agent. Upon entering the body, lomustine requires hepatic activation to form active intermediates that can modify purine bases in DNA. This modification inhibits DNA, RNA, and protein synthesis, ultimately inducing programmed cell death in rapidly dividing cells (National Institute of Diabetes and Digestive and Kidney Diseases, 2012; Guo et al., 2021). Temozolomide is an alkylating prodrug and a chemotherapeutic agent that can cross the blood-brain barrier. The brain tumor microenvironment is typically alkaline, rendering temozolomide unstable. Temozolomide spontaneously decomposes to form an active metabolite that methylates purine bases in DNA, thereby causing cellular damage and ultimately inducing apoptosis (Zhou et al., 2022; Zhang et al., 2012). Both drugs are extensively utilized in clinical practice for the radiotherapy treatment of tumors. Data from clinical trials have demonstrated that the combination of lomustine and temozolomide is more effective than either drug alone in patients with glioblastoma, resulting in prolonged average survival (Herrlinger et al., 2019). However, despite the achievements of lomustine and temozolomide in clinical settings, their adverse drug reactions (ADRs) (refer to adverse reactions that are directly induced by the drug) must not be overlooked. Adverse reactions not only affect the patient’s treatment experience but may also limit the dosage and efficacy of the drugs, potentially leading to treatment discontinuation. Therefore, a comprehensive and thorough analysis of the adverse reactions associated with these drugs is crucial for optimizing treatment regimens and enhancing treatment safety and tolerability. The existing literature primarily focuses on animal studies, mechanisms of action, and adverse events in small clinical trial samples of lomustine and temozolomide. However, no studies have yet analyzed the combined safety profile of lomustine and temozolomide using large-scale, real-world data. The present study aims to systematically analyze and compare the ADRs related to lomustine and temozolomide as reported in the World Health Organization (WHO) VigiAcess database, with the goal of identifying the antiglioma drug with the lowest risk for individualized use in clinical patients, thereby providing a valuable reference for clinical treatment.

Despite the thoroughness of pre-marketing clinical trials, the safety of these medications remains partially undefined based on data from pre-authorization studies, as these trials are conducted under controlled conditions that differ from everyday practice (Gagliardi et al., 2022). Lomustine and temozolomide, which have been commercially available for a significant period, serve a large patient demographic and have multiple applications. Therefore, it is particularly important and insightful to conduct safety research utilizing extensive data from real-world scenarios. Consequently, a more detailed characterization of ADRs linked to anti-glioma drugs is essential, leveraging spontaneous reports from pharmacovigilance databases. It is noteworthy that there is a lack of comparative studies examining the similarities and disparities in ADRs caused by these drugs. Since 2015, data archived in VigiBase has been made publicly accessible through VigiAccess (Watson et al., 2018; Habarugira and Figueras, 2021).

The VigiAccess database enables searches using the trade names of drugs, while also identifying the active ingredients and presenting the corresponding results of ADR reports. This research primarily examines two anti-glioma drugs used for treating glioma: lomustine and temozolomide. Clinicians frequently need to tailor treatment choices considering the potential risk of adverse events for each patient. To assess the occurrence of adverse reactions associated with these two drugs, we performed a descriptive study that analyzed spontaneously reported adverse reactions in the VigiAccess database and compared the rates of adverse reactions linked to these two medications.

## Materials and methods

### Drug sample

Table 1 presents the general information regarding the two anti-glioma drugs available for clinical treatment in our study.

**TABLE 1:** General information of two anti-glioma drugs.

Drug name	Brand names	Chemical formula	Prescription informaiton	Main conditions	The earliest year on the market
Lomustine	Ceenu, Gleostine	C_9_H_16_ClN_3_O_2_	taken once every 6 weeks	primary and metastatic brain tumors, refractory or relapsed Hodgkin’s disease, lung cancer	1976
Temozolomide	Temodar, Temomedac	C_6_H_6_N_6_O_2_	taken at 75 mg/M2 every day for 6 weeks along with radiation therapy; then 5 days every 4 weeks for 6 cycles	glioblastoma multiforme and refractory anaplastic astrocytoma	1999

Lomustine is likely the second most widely used drug for glioma treatment, following temozolomide. Also known as CCNU (chloroethyl-cyclohexyl-nitrosourea), lomustine is an alkylating agent that belongs to the nitrosourea family. It functions as a monofunctional alkylating agent, capable of alkylating both DNA and RNA, and can induce cross-linking of DNA, thereby acting in both cell cycle-dependent and -independent manners. A significant lesion induced by lomustine is the formation of O6-chloroethylguanine, which can be reversed by O6-methylguanine DNA methyltransferase (MGMT) (Strobel et al., 2019). Additionally, lomustine may inhibit enzymatic functions through the carbamoylation of amino acids; however, the clinical significance of this activity remains unclear. As a lipid-soluble drug, lomustine effectively permeates the blood-brain barrier, making it a suitable candidate for the chemotherapy of intrinsic brain tumors (de Gooijer et al., 2018).

Temozolomide is an imidazole tetrazine prodrug that remains stable at acidic pH and undergoes spontaneous non-enzymatic hydrolysis at neutral or slightly alkaline pH. It can be administered both orally and intravenously. Following absorption, it is rapidly converted into the active compound 5-(3-methyltriazen-1-yl) imidazole-4-carboxamide (MTIC) through non-enzymatic pathways. MTIC subsequently reacts with water to yield 5-aminoimidazole-4-carboxylamine (AIC) and the highly reactive methyldiazonium cation. These methyldiazonium cations are notably reactive and can methylate the adenine and guanine bases in DNA, primarily at the O6 and N7 positions of guanine. The repair of O6-methylguanine (O6-MeG) is facilitated by MGMT (Lips and Kaina, 2001). When MGMT activity is diminished or absent, O6-MeG mispairs with thymine, thereby activating the DNA mismatch repair (MMR) pathway (Zhang et al., 2012; Strobel et al., 2019). This activation may lead to ineffective repair cycles, resulting in DNA chain breaks and ultimately cell apoptosis.

### Data sources

Despite challenges such as incomplete data, misinformation, delayed reporting, and regional concentration of reports, spontaneous reporting systems remain a valuable source for obtaining real-world data on drug and vaccine safety, comparing treatment regimens, and elucidating the underlying mechanisms of ADRs (Hazell and Shakir, 2006). The WHO-VigiAccess database was searched on 8 November 2024, to collect all documented adverse events following the introduction of two anti-glioma drugs. The access URL is https://www.vigiaccess.org. All pharmaceutical agents under study were identified using their generic names. Data collection encompassed various age ranges, genders, years of reporting, and geographic regions, as detailed by WHO-VigiAccess. Descriptive statistics were computed using Excel 2021.

WHO-VigiAccess serves as an open-access portal to the PIDM database, facilitating the retrieval of safety reports concerning medicinal products provided by the UMC. The evaluation relied on system organ class (SOC) and preferred terms (PTs) as defined by the Medical Dictionary for Regulatory Activities (MedDRA). Consequently, records for each drug were compiled, and all distinct adverse events (AEs) (refer to all adverse reactions that occur after taking a drug) identified at the MedDRA SOC and PT levels were specified to delineate the range of toxicities. The reporting terms utilized in MedDRA were gathered from various dictionaries, including the WHO Adverse Reaction Terminology (WHO-ART) and others (Sultana et al., 2020). In total, 27 items were categorized by SOC. This research focused on the PTs, which represent the extent of publicly available information in the VigiBase database through WHO-VigiAccess. To assess the results of the identified safety signals, we organized them using outcome codes, culminating in three critical categories: death, hospitalization, and major events, which encompass life-threatening occurrences, disabilities, and congenital anomalies.

### Statistical analysis

Statistical Evaluation: A retrospective quantitative approach was adopted for this study. Descriptive analysis was conducted using Excel to assess the characteristics of victims who experienced adverse reactions from the two medications. The rate of ADR reporting for each medication was determined by dividing the number of ADR symptoms associated with that specific drug by the total number of ADR reports. The common ADRs linked to each medication were identified as the symptoms corresponding to the top 20 ADR report rates. Following this, the reported ADR symptoms for each drug were calculated, and a descriptive comparative analysis was performed. Frequencies and percentages were utilized to classify the descriptive variables.

## Results

### Description of the studied cases

The initial documentation of negative reactions to lomustine and temozolomide was recorded in the WHO-VigiAccess database in 1976 and 1997, respectively. As of 2024, the WHO has accumulated a total of 1,647 and 21,207 reports of ADRs for these two medications, resulting in an aggregate of 22,854 reports. Among these 22,854 ADR reports associated with the two anti-glioma drugs, as detailed in Table 1, there were 2,445 instances where the sex of the subjects was not specified. Notably, the number of ADR reports from men (10,100) was approximately equal to that of women (9,980), yielding a female-to-male ratio of nearly 1:1, which indicates a relatively balanced distribution. Excluding reports that lacked age information, the demographic groups with the highest rates of reported incidents were primarily those aged between 45 and 64 years. Furthermore, the majority of adverse events were reported from the Americas, accounting for 62.10% of the overall total. Table 2 provides additional details regarding the reporting years for each of the medications analyzed.

**TABLE 2:** Characteristics of ADR reports of two anti-glioma drugs.

	Lomustine	Temozolomide
Number of ADR reports	1,647	21,207
Female	626 (38.01%)	8,939 (42.15%)
Male	883 (53.61%)	9,961 (46.97%)
Unknown	138 (8.38%)	2,307 (10.88%)
0–27 days	——	9 (0.04%)
28 days to 23 months	4 (0.24%)	83 (0.39%)
2–11 years	79 (4.80%)	712 (3.36%)
12–17 years	58 (3.52%)	409 (1.93%)
18–44 years	316 (19.19%)	3,042 (14.34%)
45–64 years	564 (34.24%)	6,804 (32.08%)
65–74 years	218 (13.24%)	3,117 (14.70%)
≥75 years	43 (2.61%)	1,155 (5.45%)
Unknown	365 (22.16%)	5,876 (27.71%)
Africa	1 (0.06%)	86 (0.41%)
Americas	829 (50.33%)	13,363 (63.01%)
Asia	182 (11.05%)	2,626 (12.38%)
Europe	615 (37.34%)	4,872 (22.97%)
Oceania	20 (1.21%)	260 (1.23%)
Before 2010	362 (21.98%)	3,732 (17.60%)
2011	45 (2.73%)	702 (3.31%)
2012	14 (0.85%)	457 (2.15%)
2013	19 (1.15%)	554 (2.61%)
2014	56 (3.40%)	1,486 (7.01%)
2015	77 (4.68%)	1,074 (5.06%)
2016	83 (5.04%)	1,020 (4.81%)
2017	112 (6.80%)	1,315 (6.20%)
2018	119 (7.23%)	1,585 (7.47%)
2019	166 (10.08%)	1914 (9.03%)
2020	111 (6.74%)	1,431 (6.75%)
2021	116 (7.04%)	1,444 (6.81%)
2022	122 (7.41%)	1,463 (6.90%)
2023	121 (7.35%)	1,658 (7.82%)
2024	124 (7.53%)	1,372 (6.47%)

### Distribution of 27 SOCs of two anti-glioma drugs

Table 3 presents the reporting frequencies of 27 SOCs associated with two anti-glioma drugs. Lomustine exhibits a higher reporting rate for blood and lymphatic system disorders, gastrointestinal disorders, and hepatobiliary disorders. Conversely, Temozolomide shows a higher reporting rate for general disorders and administration site conditions, nervous system disorders, and skin and subcutaneous tissue disorders. Furthermore, the number of ADRs exceeding 10% within each SOC was eight for lomustine and nine for temozolomide.

**TABLE 3:** ADR number and report rate of 27 SOCs of two anti-glioma drugs.

System organ classes	Lomustine (N = 1,647)	Temozolomide (N = 21,207)
Blood and lymphatic system disorders	620 (37.64%)	6,749 (31.82%)
Cardiac disorders	45 (2.73%)	531 (2.50%)
Congenital, familial and genetic disorders	3 (0.18%)	169 (0.80%)
Ear and labyrinth disorders	24 (1.46%%)	112 (0.53%)
Endocrine disorders	13 (0.79%)	128 (0.60%)
Eye disorders	35 (2.13%)	316 (1.49%)
Gastrointestinal disorders	463 (28.11%)	5,151 (24.29%)
General disorders and administration site conditions	530 (32.18%)	8,295 (39.11%)
Hepatobiliary disorders	151 (9.17%)	926 (4.37%)
Immune system disorders	7 (0.43%)	247 (1.16%)
Infections and infestations	212 (12.87%)	2,950 (13.91%)
Injury poisoning and procedural complications	233 (14.15%)	3,716 (17.52%)
Investigations	385 (23.38%)	4,470 (21.08%)
Metabolism and nutrition disorders	89 (5.40%)	1,549 (7.30%)
Musculoskeletal and connective tissue disorders	49 (2.98%)	754 (3.56%)
Neoplasms benign, malignant and unspecified (including cysts and polyps)	260 (15.79%)	2,693 (12.70%)
Nervous system disorders	277 (16.82%)	4,770 (22.49%)
Pregnancy puerperium and perinatal conditions	3 (0.18%)	48 (0.23%)
Product issues	4 (0.24%)	142 (0.67%)
Psychiatric disorders	57 (3.46%)	1,030 (4.86%)
Renal and urinary disorders	50 (3.04%)	574 (2.71%)
Reproductive system and breast disorders	2 (0.12%)	65 (0.31%)
Respiratory, thoracic and mediastinal disorders	136 (8.26%)	1915 (9.03%)
Skin and subcutaneous tissue disorders	137 (8.32%)	2,213 (10.44%)
Social circumstances	6 (0.36%)	95 (0.45%)
Surgical and medical procedures	64 (3.89%)	990 (4.67%)
Vascular disorders	71 (4.31%)	1,060 (5.00%)

The five most common types of AEs related to anti-glioma drugs are as follows: general disorders and administration site conditions (8,825 cases, 38.61%), blood and lymphatic system disorders (7,369 cases, 32.24%), gastrointestinal disorders (5,614 cases, 24.56%), nervous system disorders (5,047 cases, 22.08%), and investigations (4,855 cases, 21.24%).

### Most common ADRs of two anti-glioma drugs

Table 4 presents the 20 most frequently reported ADRs associated with the two drugs. The manifestations listed are preferred terms categorized within the SOC. The commonly observed ADRs for both anti-glioma drugs include thrombocytopenia, vomiting, death, nausea, leukopenia, decreased platelet count, neutropenia, disease progression, seizures, fatigue, malignant neoplasm progression, pancytopenia, diarrhea, off-label use, pyrexia, use in unapproved indications, and drug ineffectiveness.

**TABLE 4:** Top 20 ADRs of two anti-glioma drugs.

Lomustine (N = 1,647)		Temozolomide (N = 21,207)	
ADR	Report rate %	ADR	Report rate %
Thrombocytopenia	11.35%	Thrombocytopenia	9.40%
Vomiting	9.59%	Death	7.18%
Death	9.05%	Nausea	7.04%
Nausea	6.86%	Disease progression	6.17%
Leukopenia	6.13%	Vomiting	5.13%
Platelet count decreased	4.19%	Product use in unapproved indication	4.61%
Neutropenia	4.01%	Fatigue	4.06%
Anaemia	3.89%	Off label use	4.00%
Disease progression	3.76%	Neutropenia	3.95%
Seizure	3.40%	Seizure	3.61%
Fatigue	3.34%	Pancytopenia	3.59%
Malignant neoplasm progression	3.34%	Drug ineffective	3.41%
Pancytopenia	3.28%	Platelet count decreased	3.15%
Diarrhoea	2.49%	Pyrexia	2.64%
Off label use	2.43%	Asthenia	2.52%
Pyrexia	2.31%	Malignant neoplasm progression	2.51%
Pneumonia	2.13%	Leukopenia	2.43%
Product use in unapproved indication	2.00%	Myelosuppression	2.33%
Weight decreased	2.00%	Diarrhoea	2.31%
Drug ineffective	1.88%	Rash	2.23%

Lomustine and temozolomide exhibit certain similarities in their adverse reactions. Both drugs significantly affect the hematological system and gastrointestinal tract, and they are associated with disease progression and mortality. However, notable differences exist in the incidence of specific adverse reactions: Lomustine is more likely to cause thrombocytopenia, while temozolomide is associated with a higher incidence of nausea and vomiting. Furthermore, off-label use and applications for unapproved indications were frequently noted in reports for both drugs, suggesting potential issues with appropriate use and the risks associated with their administration outside the approved scope.

### Serious AEs of two anti-glioma drugs

**FIGURE 1:**
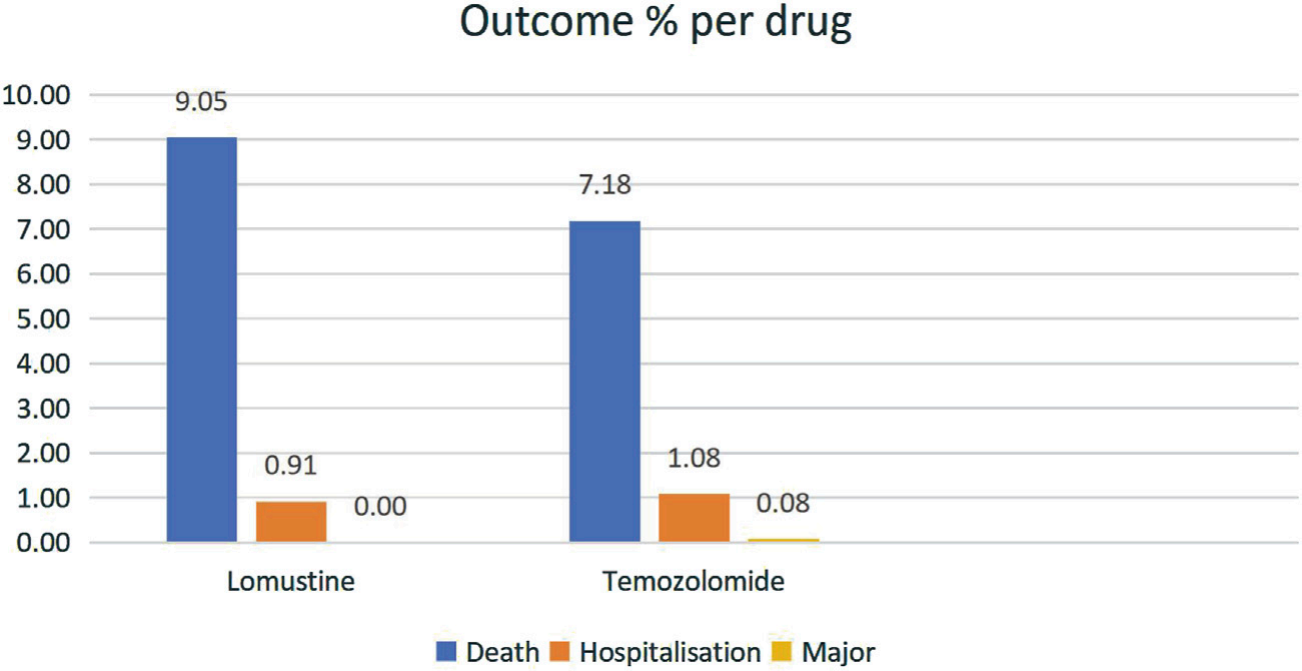
The proportions of serious adverse reactions reported for lomustine and temozolomide.

Through WHO-VigiAccess, we can identify significant adverse events associated with anti-glioma drugs, including life-threatening occurrences, disabilities, and congenital malformations. The proportions of serious adverse reactions reported for lomustine and temozolomide were 9.96% and 8.34%, respectively (Figure 1).

### The same and different points of common ADRs of two anti-glioma drugs

By examining the top 20 ADRs associated with each anti-glioma drug within the SOCs, a cumulative total of 214 identical signals was identified across the two anti-glioma drugs. All overlapping signals are detailed in Table 5. General disorders and administration site conditions emerged as the SOC with the largest number of adverse signals, with the five most frequently reported reactions being chills, no adverse event, treatment failure, asthenia, and condition aggravated. Following this, nervous system disorders ranked as the second most prevalent SOC, featuring the top five reactions of ataxia, cognitive disorder, cerebrovascular accident, speech disorder, and cerebral hemorrhage.

**TABLE 5:** Same ADRs between two anti-glioma drugs.

System organ classes	ADRS	Signal N
Blood and lymphatic system disorders	Haematotoxicity, Lymphopenia, Thrombocytopenia, Bone marrow failure, Aplastic anaemia, Neutropenia, Anaemia, Pancytopenia, Cytopenia, Agranulocytosis, Granulocytopenia, Myelosuppression, Febrile neutropenia, Leukopenia	14
Cardiac disorders	Arrhythmia, Atrial fibrillation, Myocardial infarction	3
Eye disorders	Visual impairment, Vision blurred	2
Gastrointestinal disorders	Abdominal pain, Colitis, Dyspepsia, Large intestine perforation, Vomiting, Abdominal discomfort, Abdominal distension, Stomatitis, Constipation, Rectal haemorrhage, Diarrhoea, Gastrointestinal haemorrhage, Dysphagia, Nausea, Abdominal pain upper	15
General disorders and administration site conditions	Chills, No adverse event, Treatment failure, Asthenia, Condition aggravated, Multiple organ dysfunction syndrome, Death, Malaise, Pyrexia, Ill-defined disorder, Chest pain, Oedema, Oedema peripheral, Unevaluable event, Swelling, Therapy non-responder, Disease progression, Drug resistance, Pain, Drug ineffective, Mucosal inflammation, Gait disturbance, General physical health deterioration, Fatigue, Disease recurrence	25
Hepatobiliary disorders	Cholestasis, Hepatic failure, Hepatotoxicity, Liver injury, Hepatic cytolysis, Hepatic function abnormal, Hepatitis, Liver disorder, Hyperbilirubinaemia, Jaundice	10
Immune system disorders	Hypersensitivity	1
Infections and infestations	Cellulitis, Nasopharyngitis, Oral candidiasis, Diverticulitis, *Candida* infection, Wound infection, Urinary tract infection, Infection, Septic shock, Sepsis, Pneumonia, Herpes zoster	12
Injury, poisoning and procedural complications	Incorrect dose administered, Product use in unapproved indication, Product dose omission issue, Product dispensing error, Off label use, Inappropriate schedule of product administration, Contusion, Accidental overdose, Head injury, Product use issue, Toxicity to various agents, Fall, Overdose, Medication error	14
Investigations	Aspartate aminotransferase increased, Neutrophil count decreased, Transaminases increased, Blood creatinine increased, Red blood cell count decreased, Blood pressure increased, Alanine aminotransferase increased, Liver function test abnormal, Blood lactate dehydrogenase increased, Full blood count abnormal, Platelet count decreased, Gamma-glutamyltransferase increased, Blood bilirubin increased, Full blood count decreased, Lymphocyte count decreased, Blood glucose increased, Blood alkaline phosphatase increased, White blood cell count decreased, Haemoglobin decreased, Weight increased, Weight decreased, Hepatic enzyme increased	22
Metabolism and nutrition disorders	Hypokalaemia, Hypophagia, Decreased appetite, Hypoalbuminaemia, Hyponatraemia, Dehydration	6
Musculoskeletal and connective tissue disorders	Arthralgia, Pain in extremity, Back pain, Myalgia, Muscular weakness	5
Neoplasms benign, malignant and unspecified (incl cysts and polyps)	Neoplasm recurrence, Malignant neoplasm progression, Brain neoplasm, Metastatic malignant melanoma, Tumour haemorrhage, Brain neoplasm malignant, Tumour pseudoprogression, Neoplasm progression, Neoplasm malignant, Metastases to lung, Acute myeloid leukaemia, Astrocytoma malignant, Glioblastoma, Glioblastoma multiforme, Myelodysplastic syndrome, Neoplasm	16
Nervous system disorders	Ataxia, Cognitive disorder, Cerebrovascular accident, Speech disorder, Cerebral haemorrhage, Memory impairment, Hydrocephalus, Dizziness, Hemiparesis, Generalised tonic-clonic seizure, Seizure, Paraesthesia, Headache, Depressed level of consciousness, Lethargy, Encephalopathy, Hypoaesthesia, Aphasia, Balance disorder, Neuropathy peripheral, Amnesia, Haemorrhage intracranial, Dysarthria, Brain oedema	24
Psychiatric disorders	Mental status changes, Insomnia, Confusional state, Depression, Disorientation	5
Renal and urinary disorders	Renal impairment, Acute kidney injury, Proteinuria, Renal failure	4
Respiratory, thoracic and mediastinal disorders	Dyspnoea, Epistaxis, Respiratory failure, Pulmonary embolism, Hypoxia, Acute respiratory distress syndrome, Respiratory distress, Pleural effusion, Cough, Pneumonitis, Interstitial lung disease, Lung disorder, Pulmonary thrombosis	13
Skin and subcutaneous tissue disorders	Urticaria, Rash maculo-papular, Rash, Alopecia, Toxic epidermal necrolysis, Rash erythematous, Dry skin, Pruritus, Erythema, Purpura, Petechiae, Rash pruritic	12
Surgical and medical procedures	Hospitalisation, Therapy interrupted, Therapy change, Therapy cessation, Hospice care	5
Vascular disorders	Haemorrhage, Deep vein thrombosis, Hypertension, Thrombosis, Hypotension, Embolism	6

When comparing the top 20 ADRs reported by the two anti-glioma drugs, there are 25 differences at the PTs level (Table 6). Among these, the two drugs exhibit the highest number of infections and infestations, totaling 25. The top five adverse reactions reported by lomustine include *mycobacterium chelonae* infection, influenza, bacteraemia, oral fungal infection, and *pseudomonas* infection. In contrast, the top five adverse reactions reported by temozolomide are pneumocystis jirovecii infection, meningitis, herpes simplex encephalitis, neutropenic sepsis, and pneumocystis jirovecii pneumonia. Furthermore, regarding neoplasms—benign, malignant, and unspecified (including cysts and polyps)—lomustine reports the top five adverse reactions as non-Hodgkin’s lymphoma, basal cell carcinoma, acute myelomonocytic leukaemia, chronic myelomonocytic leukaemia, and myelofibrosis, while temozolomide reports its top five adverse reactions as metastases to the central nervous system, metastases to bone, tumour necrosis, malignant melanoma, and metastases to the liver.

**TABLE 6:** Different ADRs bwtween two anti-glioma drugs.

System organ classes	Lomustine	Temozolomide
Blood and lymphatic system disorders	Hypochromic anaemia, Thrombotic microangiopathy, Leukocytosis, Febrile bone marrow aplasia, Haemolytic anaemia, Platelet disorder, White blood cell disorder	Eosinophilia, Immune thrombocytopenia
Cardiac disorders	Left ventricular dysfunction, Cardiomyopathy, Cardiac failure, Pericardial effusion, Tricuspid valve incompetence, Cardiomegaly, Mitral valve incompetence	Tachycardia, Cardiac arrest
Congenital, familial and genetic disorders		Hypermutation
Ear and labyrinth disorders	Ototoxicity, Deafness, Deafness neurosensory, Deafness unilateral, Tinnitus	Vertigo
Endocrine disorders	Hypothyroidism, Precocious puberty	Diabetes insipidus
Eye disorders	Blindness, Diplopia, Optic atrophy, Visual field defect	
Gastrointestinal disorders	Flatulence, Hyperaesthesia teeth, Pancreatitis acute, Gastric haemorrhage, Enteritis, Gastrointestinal perforation, Haematemesis, Melaena, Gastrointestinal necrosis, Gastritis, Intestinal perforation, Mouth ulceration, Dry mouth	Gastrointestinal disorder, Pancreatitis, Ascites
General disorders and administration site conditions	Face oedema, Adverse drug reaction, Impaired healing, Exercise tolerance decreased, Therapeutic product effect decreased	Drug interaction, Peripheral swelling, Adverse event, Drug intolerance, Gait inability
Hepatobiliary disorders	Hepatic steatosis, Hepatocellular injury, Jaundice cholestatic, Mixed liver injury, Cholestatic liver injury, Hepatitis cholestatic, Hypertransaminasaemia, Venoocclusive liver disease, Acute hepatic failure, Hepatitis acute	Drug-induced liver injury
Immune system disorders	Graft versus host disease	Drug hypersensitivity
Infections and infestations	*Mycobacterium chelonae* infection, Influenza, Bacteraemia, Oral fungal infection, *Pseudomonas* infection, Myelitis, Gastrointestinal infection, COVID-19, Geotrichum infection, Ecthyma, Meningitis cryptococcal, *Aeromonas* infection, Bronchopulmonary aspergillosis, Respiratory tract infection, Necrotising fasciitis, Viral infection, Pneumonia bacterial	Pneumocystis jirovecii infection, Meningitis, Herpes simplex encephalitis, Neutropenic sepsis, Pneumocystis jirovecii pneumonia, Aspergillus infection, Staphylococcal infection, Pneumonia aspiration
Injury, poisoning and procedural complications	Prescribed underdose, Product administration error, Product prescribing error, Wrong product administered, Infusion related reaction, Circumstance or information capable of leading to medication error, Accidental exposure to product by child	Radiation necrosis, Product prescribing issue, Subdural haematoma, Product administered to patient of inappropriate age, Wrong technique in product usage process
Investigations	Blood test abnormal, Red cell distribution width increased, Mean cell haemoglobin increased, Mean cell volume increased, Heart rate decreased, Body temperature decreased, Oxygen saturation decreased, Liver function test increased	Haematocrit decreased, Blood potassium decreased, Heart rate increased, Magnetic resonance imaging head abnormal, C-reactive protein increased, White blood cell count increased
Metabolism and nutrition disorders	Cachexia, Hypocalcaemia, Fluid intake reduced, Failure to thrive, Tumour lysis syndrome, Type 2 diabetes mellitus, Lactic acidosis, Hypermagnesaemia, Malnutrition, Hypophosphataemia	Hyperglycaemia
Musculoskeletal and connective tissue disorders	Mobility decreased, Muscle spasms	Bone pain
Neoplasms benign, malignant and unspecified (incl cysts and polyps)	Non-hodgkin’s lymphoma, Basal cell carcinoma, Acute myelomonocytic leukaemia, Chronic myelomonocytic leukaemia, Myelofibrosis, Leukaemia, Myeloid leukaemia, Ganglioglioma, Leukaemia granulocytic, Kaposi’s sarcoma, Second primary malignancy, Acute leukaemia	Metastases to central nervous system, Metastases to bone, Tumour necrosis, Malignant melanoma, Metastases to liver, Recurrent cancer
Nervous system disorders	Coordination abnormal, Transient ischaemic attack, Migraine, Polyneuropathy, Cerebrovascular disorder, Burning sensation	Tremor, Epilepsy, Dysgeusia, Syncope, Loss of consciousness, Somnolence
Product issues	Manufacturing product shipping issue	Product substitution issue
Psychiatric disorders	Mania, Dysphemia, Anger	Anxiety, Agitation
Renal and urinary disorders	Tubulointerstitial nephritis, Anuria, Incontinence, Urine abnormality, Pollakiuria	Urinary incontinence
Respiratory, thoracic and mediastinal disorders	Oropharyngeal pain, Dysphonia, Pulmonary toxicity, Oropharyngeal discomfort, Respiratory disorder, Pulmonary fibrosis, Acute respiratory failure, Dyspnoea exertional	Lung infiltration, Pulmonary oedema, Atelectasis
Skin and subcutaneous tissue disorders	Blister, Skin irritation, Skin disorder, Toxic skin eruption, Rash macular, Rash papular, Acne	Stevens-johnson syndrome, Palmar-plantar erythrodysaesthesia syndrome, Dermatitis, Drug eruption, Drug reaction with eosinophilia and systemic symptoms
Social circumstances	Refusal of treatment by patient	
Surgical and medical procedures	Platelet transfusion	Surgery, Brain operation
Vascular disorders	Poor venous access, Ischaemia, Blood pressure fluctuation, Venous thrombosis, Shock	Haematoma

## Discussion

The Spontaneous Reporting System (SRS) is widely employed in pharmacovigilance to assess the safety of suspected adverse events. Clinical trials are subject to limitations, including rigorous designs, stringent enrollment criteria, restricted sample sizes, and short follow-up durations, which may hinder the accurate reflection of real-world scenarios characterized by diverse patient demographics and comorbidities. Consequently, data derived from the SRS database can more effectively illustrate the safety of specific drugs in real-world settings and plays a crucial role in signal detection. Currently, research on the safety signals of numerous drugs primarily relies on three key databases: the EudraVigilance Data Analysis System (EVDAS), the Food and Drug Administration (FDA) Adverse Event Reporting System (FAERS), and WHO-VigiBase^®^ (Vogel et al., 2020). In 2015, the WHO introduced WHO-VigiAccess, which provides public access to the information contained in VigiBase^®^, the WHO’s global repository of reported potential adverse effects associated with medicinal products. Analyzing data from the WHO-VigiAccess database can reveal previously unknown associations between drugs and AEs, as well as validate some established clinical connections (Yamoah et al., 2022). This study aims to evaluate the post-market adverse events linked to two anti-glioma drugs using the WHO-VigiAccess database.

According to data from WHO-VigiAccess, 62.10% of adverse events related to these two anti-glioma drugs were reported from the Americas, followed by Europe, while Africa reported the lowest incidence of adverse events. Prior research has highlighted a significant issue regarding the low reporting rates of adverse events in both Africa and Oceania (Gidudu et al., 2020; Alawadhi et al., 2012). In South Africa, a shortage of medical understanding concerning biopharmaceuticals among healthcare workers, coupled with high costs and complicated procurement procedures, further exacerbates the challenges associated with the use of these medications (Gidudu et al., 2020; Martelli et al., 2017; Kvamme et al., 2020). The African region has been noted for having the lowest incidence of reported adverse events, which may be attributed to insufficient social mobilization, restricted access to adverse event reporting mechanisms, and low levels of information system coverage.

The number of ADR reports from men (10,100) was approximately equal to that of women (9,980), resulting in a female-to-male ratio of nearly 1:1, which indicates a relatively balanced distribution. When excluding reports lacking information on age, the demographic groups with the highest rates of reported incidents were primarily those aged between 45 and 64 years. An AE)with a reporting rate of 1% or greater is generally considered common (Chen et al., 2019). Major adverse events associated with anti-glioma drugs include life-threatening incidents, disabilities, and congenital malformations. The mortality rates for lomustine and temozolomide are 9.96% and 8.34%, respectively. Lomustine exhibits a higher reporting rate for blood and lymphatic system disorders, gastrointestinal disorders, and hepatobiliary disorders. Conversely, temozolomide has a higher reporting rate for general disorders and administration site conditions, as well as nervous system disorders and skin and subcutaneous tissue disorders. Through the VigiAccess database, we identified the top five adverse reactions related to lomustine: thrombocytopenia (11.35%), vomiting (9.59%), death (9.05%), nausea (6.86%), and leukopenia (6.13%). For temozolomide, the top five adverse reactions are thrombocytopenia (9.40%), death (7.18%), nausea (7.04%), disease progression (6.17%), and vomiting (5.13%). The frequently observed ADRs for both anti-glioma drugs include thrombocytopenia, vomiting, death, nausea, leukopenia, decreased platelet count, neutropenia, disease progression, seizures, fatigue, malignant neoplasm progression, pancytopenia, diarrhea, off-label use, pyrexia, product use in unapproved indications, and drug ineffectiveness.

Lomustine is an emetogenic chemotherapy drug that typically necessitates standard antiemetic precautions, which are generally effective. Thrombocytopenia is the primary toxic reaction associated with this treatment, often leading to dosage reductions, delays in chemotherapy cycles, or even cessation of therapy. Neutropenia and lymphopenia occur less frequently and are generally less severe. Despite this toxic profile, the development of myelodysplastic syndromes and leukemias as sequelae of lomustine chemotherapy is rare. This rarity is presumably due to the limited life expectancy of glioma patients, which decreases the likelihood of complications arising years after exposure to the drug (Czarnywojtek et al., 2023).

The adverse reactions associated with temozolomide are typically classified as NCI common toxicity criteria (CTC) grade 1 or 2 (mild to moderate) and are generally self-limiting. Nausea and vomiting can be effectively managed with antiemetics. The incidence of severe nausea and vomiting (CTC grade 3 or 4) has been reported at 10% and 6%, respectively (Matsuda et al., 2015; Bae et al., 2014). The primary dose-limiting toxicity of temozolomide is myelosuppression, which can occur at any dose but tends to be more pronounced at higher doses (Ortiz et al., 2021). Patients receiving higher doses have experienced adverse reactions such as severe and prolonged myelosuppression, infections, and, in some cases, death. Patients who experience an overdose should have their complete blood counts monitored and receive supportive care as necessary.

Undoubtedly, this study has certain limitations. Firstly, the voluntary nature of the spontaneous reporting system leads to issues such as irregular recording, incorrect reporting, delayed reporting, and incomplete data, all of which complicate data analysis and affect the results. For example, this study lacks data on gender, age, and region, and there is a notable absence of reports from Africa. Therefore, the conclusions and data presented here may not be generalizable to all populations. Secondly, the WHO-VigiAccess database contains cumulative data for drugs since their market introduction, but it does not provide annual ADR data. The 13-year difference in market introduction between the two drugs limits our ability to conduct a more in-depth analysis of the data. In addition, the relatively short life expectancy associated with glioma often precludes the opportunity to observe long-term complications that may arise after several years of drug treatment.

The WHO-VigiAccess database, which operates on a voluntary basis for adverse event reporting, presents several challenges that hinder its ability to provide a complete and thorough count of adverse events. The database may lack essential information regarding reported incidents, highlighting the need to enhance the transparency of reporting practices. By improving the clarity and accessibility of the data available to the public, stakeholders can engage in more effective screening for potential connections between pharmaceuticals and adverse reactions. This enhancement would also help mitigate misguidance that could arise from incomplete or unclear information. The reliance on a spontaneous reporting system carries significant inherent limitations, primarily due to various biases that can affect the reporting process. These biases include notoriety bias, where more well-known drugs receive disproportionate attention, selection bias, which skews the data towards certain demographics, and under-reporting, which typically results in substantial gaps in data collection (Faillie, 2019). In the context of the current study, it was noted that the missing data included adverse events that could not be specifically linked to certain genders or age groups, complicating the interpretation of the data. Additionally, the cumulative nature of the VigiAccess database presents challenges in isolating ADRs on a yearly basis. When medications are introduced to the market at different times, the volume of reported ADRs can vary significantly, complicating efforts to compare signal differences across all drug classes concurrently. Consequently, conducting further data mining becomes impractical. In this analysis, the focus was placed on aggregating the number of ADRs reported over recent years and correlating these with the number of PTs associated with various drugs. This approach was designed to mitigate the impact of the differing timelines of drug market introductions on the study’s outcomes. However, the findings are limited to relative comparisons involving only the two anti-glioma drugs examined in the study.

## Data Availability

The original contributions presented in the study are included in the article/supplementary material, further inquiries can be directed to the corresponding authors.
